# Systems Biology and Biomechanical Model of Heart Failure

**DOI:** 10.2174/157340312803217238

**Published:** 2012-08

**Authors:** George E Louridas, Katerina G Lourida

**Affiliations:** a Professor of Cardiology, From: Department of Cardiology, Aristotle University, Thessaloniki, Greece; b BSc (Hons) Computing, From: Department of Cardiology, Aristotle University, Thessaloniki, Greece

**Keywords:** System biology, heart failure, wall stress, remodeling, heart failure models.

## Abstract

Heart failure is seen as a complex disease caused by a combination of a mechanical disorder, cardiac remodeling and neurohormonal activation. To define heart failure the systems biology approach integrates genes and molecules, interprets the relationship of the molecular networks with modular functional units, and explains the interaction between mechanical dysfunction and cardiac remodeling. The biomechanical model of heart failure explains satisfactorily the progression of myocardial dysfunction and the development of clinical phenotypes. The earliest mechanical changes and stresses applied in myocardial cells and/or myocardial loss or dysfunction activate left ventricular cavity remodeling and other neurohormonal regulatory mechanisms such as early release of natriuretic peptides followed by SAS and RAAS mobilization. Eventually the neurohormonal activation and the left ventricular remodeling process are leading to clinical deterioration of heart failure towards a multi-organic damage. It is hypothesized that approaching heart failure with the methodology of systems biology we promote the elucidation of its complex pathophysiology and most probably we can invent new therapeutic strategies.

## INTRODUCTION

Systems biology is a term selected for the study of the vast amount of experimental data advanced from current technologies in genomics and proteomics [1]. It is a novel area of knowledge that connects biology to other synthetic disciplines such as computer science and engineering. The goal is to synthesize complex data from various hierarchies (genome, proteome, transcriptome, metabolome) and to produce useful models explaining complex biological phenomena. The systems biology term as defined above facilitates the explanation of the principles of living systems and organisms and achieves an understanding of higher-order combinations as are various clinical phenotypes. 

The explanation given to the clinical syndrome of heart failure is limited if only the pathophysiological mechanisms are described. Also the specific clinical models developed to interpret the variety of clinical phenotypes are not sufficiently coherent to explain the progressive deterioration of heart failure and some of the successful aspects of the new therapeutic agents. Targeting the involved molecular mechanisms, only the biomechanical model predicts that the novel therapeutic strategies interrupt the viscous cycle of myocardial dysfunction and cardiac remodeling [2].

The present paper debates the biomechanical model of heart failure under the current concept of systems biology methodology exploring the connection of the complex metabolic and regulatory networks with the clinical phenotypes of heart failure. It is accepted that early (initial) mechanical stresses applied in the myocytes and/or mechanical changes in the left ventricular cavity comprise the primary initiating causes for cardiac remodeling and neurohormonal activation.

It is hypothesized that only through systems biology methodology can we explain the complex phenomenon of heart failure and defend the concept of the biomechanical model. This approach will increase the knowledge of the pathogenesis of heart failure and eventually will enable the development of effective drug therapy.

## SYSTEMS BIOLOGY APPROACH

The nature and the biological world constitute a complex system compiled of many interacting components that produce complex physical and biological phenomena. The research on complex phenomena extends from physics to biological and the medical world [3]. The physical concepts of networks with links and nodes have similar counterparts in biological systems with their metabolic networks, biochemical pathways, modular functional units and clinical phenotypes.

A network of interactions is considered modular if it is made of autonomous interconnected components that act together in performing some discrete physiological function (functional module) [4, 5]. The concept of modularity is applied therefore to some interacting cellular molecular components generating this functional unit with specific cellular functions [6]. On the other hand complex cellular and organismal function can be accomplished by connecting modules together. The modules have been evolved to perform biological functions and should be recognized as a critical level of biological organization. Also their behavior cannot be predicted by analyzing the properties of the detached components. The organisms have a modular organization involving supramolecular networks, cellular systems, regulatory systems and tissues that produce a variety of clinical phenotypes. 

Systems biology integrates diverse areas of science targeting the underlying principles of hierarchical metabolic and regulatory systems from the cell to an organismal level [7]. It entails the systematic assembly of biological and medical knowledge, from molecules to organisms, and the subsequent integration into comprehensive computer models replacing animal testing. Complex multifactorial diseases, like heart failure, are interpreted as conceptual models, with their cellular and molecular components assembled in functional modules with a faulty coordination. The methodology of systems biology defines the early mechanical stresses, the clinical functional modules and the clinical phenotypes of the heart failure syndrome.

## CLINICAL FUNCTIONAL MODULES IN HEART FAILURE

**Fig. (1):**
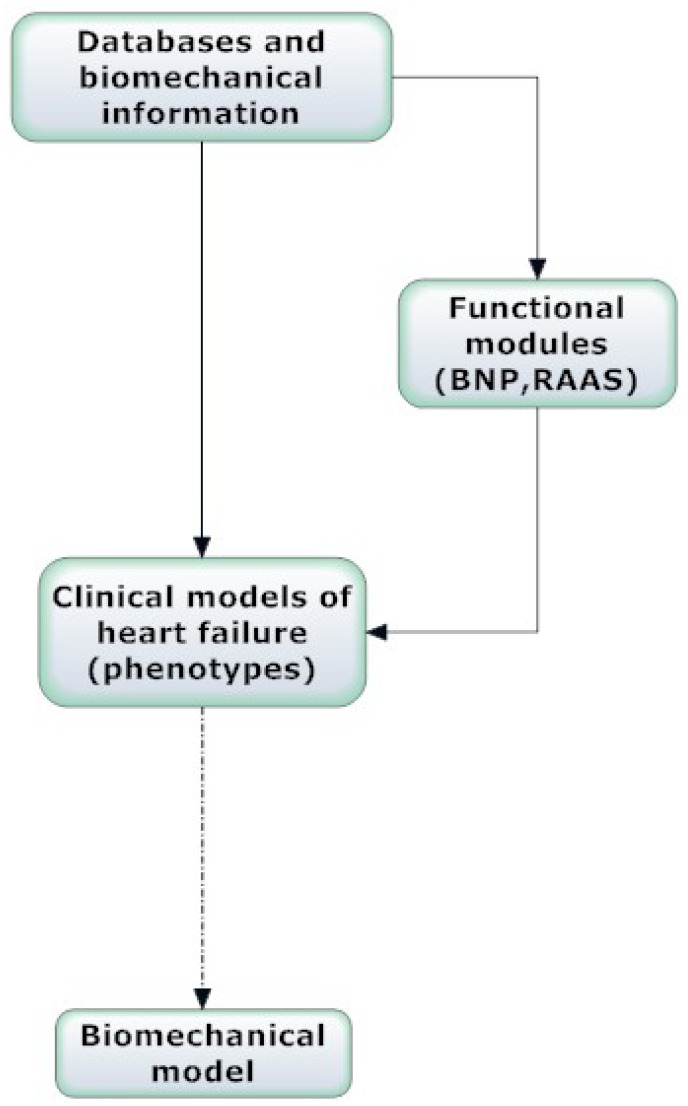
Systems biology approach and biomechanical model of heart failure.

The heart should be considered as a multifunctional organ that participates in the homeostatic regulation of the body and is an essential element of a complex and integrated network of organs and systems. Human heart failure from the point of view of systems biology is considered as a syndrome with different causes that involves many clinical appearances or clinical phenotypes. Each clinical phenotype involves many biochemical pathways, pathophysiological changes and physiological regulatory systems. These regulatory systems are important in upgrading or inhibiting the cardiac cavity remodeling process that delineates the syndrome. Two of the most important regulatory systems in heart failure, the natriuretic peptide axis and the renin-angiotensin-aldosterone system (RAAS), are considered examples of critical functional modules [8]. In the present review these regulatory systems are also referred to as functional modules, extending therefore the application of the cellular concept of module to a higher degree of organismal organization, in the level of the natriuretic peptide axis and RAAS. These systems seem to behave like functional modules selected for their ability to adapt for survival under the deleterious environment of heart failure. The methodology of systems biology applied to the above functional modules would advance further our understanding of heart failure progression (Fig. **1**). 

### a Natriuretic Peptide Axis

Atrial natriuretic peptide (ANP), brain natriuretic peptide (BNP) and their prohormones (proANP and proBNP) are secreted by cardiomyocytes. Under normal circumstances the ANP and BNP are released after increase of the atrial and myocardial stretch or after excessive sodium intake. In patients with heart failure the natriuretic peptides release is considered the earliest regulatory mechanism that is motivated in contrast to later sympatho-adrenal system (SAS) and RAAS activation. It is accepted that the ANP is produced mainly from atria and BNP from ventricles, and they are differently regulated in the two cavities. Therefore two cardiac endocrine systems are working in parallel, one in the atria producing ANP and the other in the ventricles secreting BNP [9]. In chronic cardiac dysfunction or heart failure larger amounts of BNP than ANP are synthesized and secreted due to the thickness of ventricular myocardium. The chronic activation of neurohormonal pathways in patients with heart failure represents a compensatory mechanism determined to overcome cardiac dysfunction. Moreover in chronic heart failure besides the neurohormonal overexpression there is an excessive release of endogenous vasodilatory substances (nitric oxide) and vasoconstrictive factors (angiotensin, endothelin and epinephrine). 

The BNP is considered both as a biomarker of heart failure diagnosis and as a prognostic factor in various cardiovascular diseases. In clinical practice the BNP sensitivity and specificity for increased filling pressure detection are lower than the echocardiographic estimation criteria for filling pressure. In a recent article doppler echocardiography methodology increased the accuracy of heart failure diagnosis in the presence of intermediate BNP or amino-terminal proBNP (NT-proBNP) and improved the stratification of risk across all periods of heart failure [10]. Nevertheless the BNP remains as a marker of myocyte distress and is considered a diagnostic factor in acute and acutely decompensated chronic heart failure [11]. 

The natriuretic peptides possess vasodilator, natriuretic and diuretic effects, and an inhibitory action on immunological and inflammatory systems, and on growth factors [12, 13]. They also improve the endothelial dysfunction with the decrease of the shear stress and inhibit platelet activation [14]. In patients with heart failure, the high levels of BNP with the beneficial physiological effects of vasodilation, natriuresis and increased diuresis, are not adequate to avert the disease progression to end-stage disease. In some heart failure models with an appropriate clinical context (cardiorenal dysfunction), treatment with natriuretic peptides seems reasonable as the presence of the natriuretic peptides is important and beneficial in the interactions between heart and kidney.

Systems biology approach to the natriuretic peptide axis is revealed from genomic and proteomic studies which identified many locations that were responsible for heart failure exacerbation. Genomic studies recognized multiple polymorphic structural variants of the BNP gene, which were responsible for the blood levels of BNP and NT-proBNP in normal subjects and in patients with heart failure [15, 16]. Also were identified many single-nucleotide polymorphisms (SNPs) of the gene for the enzyme corin, which is a transmembrane serine esterase responsible for the cleavage of the proBNP to active form of BNP-32. The corin gene SNPs are probably connected to human hypertension and adverse cardiac remodeling [17]. 

Recent proteomic studies define some aspects of the functional importance of the natriuretic peptide axis in comprehending the heart failure syndrome. The BNP-32 is considered to represent the main biologically active form of BNP and predictably is increased in patients with heart failure. However, using a novel method of mass spectrometry, new high molecular weight forms of BNP have recently been discovered that are less biologically active than BNP-32, but with the current assays were erroneously identified as BNP-32 or NT-Pro BNP [18]. The above investigators found these forms of high molecular weight BNP in the blood of four patients with NYHA class IV symptoms while the classical endogenous BNP-32 was absent using their method of mass spectroscopy. These additional molecular forms of BNP have reduced biological activity but could be significant from **a** diagnostic and prognostic point of view. 

The increased BNP in patients with heart failure is inadequate to delay the disease progression and its beneficial physiological effects are reduced partly because of the up-regulation of the phosphodiesterase type-5 enzyme (PDE5) [19]. The PDE5 is implicated in the breakdown of cyclic guanosine monophosphate (cGMP) which is an intracellular mediator in the phosphorylation of specific proteins and is involved in the BNP pathway. The PDE5 increase in human heart failure is implicated in the BNP inefficiency and its role should be defined.

The levels of natriuretic peptides in patients with chronic heart failure are increased compared with healthy individuals. Excessively high circulating levels of natriuretic peptides are confirmed in patients with chronic heart failure presented with fluid retention and vasoconstriction. The defective biological activity of the natriuretic peptides system suggests a kind of peripheral resistance to the biological effect of natriuretic peptides [20]. This peripheral resistance to the biological efficiency of natriuretic peptides in patients with heart failure is due to different mechanisms existing at prereceptorial, receptorial, and postreceptorial levels. In patient with heart failure the three different types of natriuretic peptide specific receptors (type-A, type-B, type-C) are inhibited by the counterregulatory systems (angiotensin II, endothelin-1, adrenergic agents). Pharmaceutical therapies with angiotensin converting enzyme inhibitors (ACE), angiotensin II-receptor antagonists, and β-blockers inhibit the action of the counterregulatory systems causing an increased natriuresis [9]. 

The above data support the hypothesis that the natriuretic peptides are elements of an integrated network that includes the cardiac muscular, endocrine, immune, and nervous system.

### b Renin- Angiotensin- Aldosterone System

The depressed left ventricular function generates a variety of physiological reactions in an endeavor to remedy the diseased myocardium. These counteracting responses that include the increased activity of both the SAS and the RAAS, are considered deleterious for the myocardium, and therefore correctly their association with the cardiovascular and cardiorenal disease became a therapeutic target. The SAS is activated early (but after the activation of BNP) in the course of heart failure due to autonomic imbalance or to loss of the inhibitory effect of the baroreceptor reflexes. The RAAS is activated later in the course of heart failure with the possible mechanisms of the renal hypoperfusion due to low cardiac output and the increased renin release due to sympathetic stimulation of the kidney.

The most significant classes of drugs targeting the RAAS are the β-adrenergic antagonists (β blockers) (reduce renin), the angiotensin-converting enzyme inhibitors (ACEI) (reduce levels of angiotensin II) and the AT_1_ receptor blockers (ARBs) ( antagonize angiotensin II binding to AT_1_ receptors). The above therapeutic agents to a degree could prevent or reverse heart failure deterioration and progression. These treatments cope with the overexpression of the biologically active neurohormonal molecules that exert deleterious effects on the heart and circulation [21]. The methodology of systems biology extends our understanding of the RAAS integrating the individual molecules of the system and their action using computer simulated models [22]. The computer simulations integrate isolated experiments with computer simulated RAAS models under physiological or diseased states. The integrated knowledge than the reductionist interpretation of the isolated experiments gives more information and understanding of the RAAS attitude towards the disease.

Systems biology approach supports the hypothesis that there are two integrated and opposing systems acting not only at the cardiac level but in the whole body. The first system is the RAAS with vasoconstrictive, sodium retaining, thrombophylic, prohypertrophic, and proinflammatory properties, while the second is the natriuretic peptide system that manifests opposing qualities such as natriuretic, vasodilating, anti-inflammatory, anti-thrombotic and anti-hypertrophic. In patients with heart failure the first system controls the hemodynamic homeostasis, initially as a counterbalancing mechanism but later induces a progressive and deleterious worsening of the cardiac function. The second opposing mechanism, the natriuretic peptide system, seems to be inadequate in delaying the heart failure progression.

## HEART FAILURE DEVELOPMENT AND BIOLOGICAL MODELS

The goal of modern clinical cardiology is to obtain a reliable description and understanding of the human physiology in various states and diseases, to incorporate the different physiological and interrelated derangements presented in databases, and finally to use the available clinical variables constructing specific clinical models (Fig. **1**).

In the present review the clinical syndrome of heart failure is discussed following the classification of the clinical model system proposed by Mann and Bristow [2]. 

### a Cardiorenal Model 

Many patients clinically are presented with a combined cardiac and renal dysfunction, usually referred to as “cardiorenal syndrome”, while renal dysfunction is considered a common and progressive complication of chronic heart failure. The co-existence or the interaction of the two clinical problems has a bad prognosis and often the primary disease of one organ precipitates secondary dysfunction of the other [23]. The combined cardiac and renal dysfunction has an underlying pathophysiology with lack of clarity and no general agreement as to its appropriate diagnosis and management [24]. The normal pathophysiological mechanisms that regulate the extracellular fluid volume by the kidney and the systemic haemodynamics by the heart are still puzzling [25]. In combined chronic renal disease and heart failure the pathophysiology is more complex involving increased activity of the RAAS, release of endogenous vasodilatory factors (natriuretic peptides, nitric oxide), oxidative stress, inflammation, and increased activity of the sympathetic nervous system with excessive production of vasoconstrictive mediators (epinephrine, angiotensin, endothelin) [26]. A new classification of the cardiorenal syndrome with five clinical subtypes was recently presented that includes the pathophysiology, the chronology of the pathophysiological interactions and the nature of the combined heart/kidney disorder [27]. The therapeutic logical strategy to use diuretics for the control of the abnormal volume status has not affected the progression of the heart failure. Notwithstanding the limited success of the present diuretic treatment the appreciation of the complex pathophysiological and clinical interactions between the two organs would provide the tools for specific treatments and interventions. The clinical understanding of the cardiorenal syndrome would help to design appropriate trials to uncover the underlying pathophysiology and the natural history of chronic heart failure, and also to identify new and effective therapies [28]. Application of systems biology concept in the elucidation of cardiorenal syndrome will help to synthesize interrelated triggers, such as oxidative stress, nitrosative stress, signal transduction, calcium handling, mitochondrial dysfunction, mutations and apoptosis with impaired cardiac pump function and secondary neurohormonal responses [29]. 

### b Cardiocirculatory or Hemodynamic Model

Soon it was realized that heart failure was associated with a compromised cardiac output, reduction in pumping capacity and significant peripheral vasoconstriction. This hemodynamic picture patterned the cardiocirculatory or hemodynamic model for heart failure [2]. In this model heart failure is considered the outcome of the diminished pumping capacity of the heart and disproportionate peripheral vasoconstriction [30]. The above argument led to the use of inotropes and vasodilators with the intention to increase cardiac output. The use of these drugs has neither prevented the worsening of the heart failure nor have they prolonged the life of the patients with severe heart failure [31]. 

### c Neurohormonal Model

The neurohormonal model contributed significantly to the development of specific drugs for heart failure therapy and followed the mainstream of clinical practice, but failed to adequately explain the progression of the disease. In the last thirty years three major classes of drugs, ACEI, ARBs and β-blockers have been introduced in the treatment of patients with heart failure and have changed our understanding of the natural history of heart failure. Experimental and clinical trials demonstrated that the above pharmaceutical intervention potentially altered the natural history of heart failure and prevented cardiac pump dysfunction and cardiac dilatation. Although both ACEI and ARBs drug classes target angiotensin II, their different mechanism of action in some biochemical interactions and receptors, produces different therapeutic effects [32]. Telmisartan was as effective as the ramipril in the prevention of cardiovascular death, myocardial infarction, stroke, or hospitalization for heart failure [33]. The term ‘neurohormonal model’ is based on the assumption that many substances presented in patients with heart failure were produced by the neuroendocrine system and affected the heart like an endocrine process. Nevertheless it was shown that the neurohormones norepinephrine and angiotensin II were acting in an autocrine and paracrine way, and the angiotensin II, tumor necrosis factor (TNF), endothelin and natriuretic peptides are growth factors (or cytokines) produced by cardiac myocytes and other cardiac cells [2]. The neurohormonal model based on experimental and clinical studies has explained well-defined clinical phenotypes which were characterized by end-organ damage and derangements of multiple biochemical pathways [34-37]. Some experimental studies with increased concentrations of neurohormones managed to create some facets of heart failure phenotype while clinical studies showed clinical improvement in patients with heart failure with drugs opposing the harmful process of neurohormones [38]. The neurohormonal model explains the classical clinical failure phenotype independent of the original cause of heart failure. The myocardial disease progression of the failing heart is motivated by the cardiac adrenergic activity and the peripheral vasoconstriction of the neurohormones with their predictable effect.

The chronic hyperadrenergic activity results in desensitization of β-receptors and reduction of the intracellular cAMP synthesis [39, 40]. The beneficial impact of β-blocking drugs in clinical trials confirms the significance of the increased chronic β-adrenergic activity with its deleterious consequence in myocardial contractility, remodeling and natural progression of heart failure. The deleterious effects are due primarily to β_1-_receptor signaling, and both β_1_-receptor blocking selective and nonselective drugs have similar beneficial clinical results. The remarkable effects of β-blocking drugs in the treatment of chronic heart failure patients are most probably due to the class effects of β_1_-receptor blockade [41, 42]. In contrast to sympatholytic agents the inhibitory effect of the β-blockers can be easily inverted by norepinephrine competition when the mobilization of the powerful adrenergic support is needed.

The above described medical therapy brings out in some of the patients stabilization of the disease but in the majority of them the progression of heart failure continues and sometimes generates hard to cure forms of neurohormonal overexpression [43] The most convincing explanation for the ineffectiveness of the neurohormonal inhibition is the existence of other metabolic pathways for neurohormones that are not affected by the present therapeutic neurohormonal antagonism. It seems that heart failure advances independently of the neurohormonal situation and therefore the neurohormonal model appears insufficient to elucidate the nature and the progression of heart failure.

### d. Biomechanical Model

In the biomechanical model the heart failure phenotype is either produced from the neurohormonal activation and progression to deleterious changes of cardiac function and remodeling, or the left ventricular deterioration is advanced directly from the left ventricular remodeling [2,44]. The term of myocardial remodeling is applied to the cellular and molecular alterations after a myocardial insult (i.e., pressure or volume overload, necrosis) ushering to a change in dimensions, shape, and function of cardiac chambers. Therefore the evolution of left ventricular remodeling is a complex procedure that involves primary the volume of both cardiac myocytes and other elements of the myocardial tissue, and afterwards the changes in geometry and architecture of the left ventricular cavity. Some experimental and clinical studies confirm the reversibility of left ventricular myocardial and cavity changes associated with the remodeling process, and reassure the reversibility of heart failure [45, 46]. Myocardial remodeling has a primarily mechanical origin with adaptive purposes that produces qualitative changes with rearrangement of normally existing structures [47] Mechanical changes taking place during left ventricular remodeling may cause deterioration of heart failure. The larger size (dilation) and the change in the geometry of left ventricle to a more spherical shape augment the mechanical load of the failing ventricle increasing the left ventricular end-diastolic wall stress, end-diastolic volume and left ventricular thinning. The increased end-diastolic wall stress causes subendocardial hypoperfusion while the wall thinning and the increased afterload result in ‘’afterload mismatch’’ [48, 49]. Both above changes are leading to an increase of oxygen utilization and to a decrease of left ventricular performance. 

Therefore left ventricular remodeling is a process that involves cardiac myocytes, extracellular myocardial matrix, geometry of left ventricular chamber, ensembles of genes and multiple molecular mechanisms. Each of these various elements of the remodeling process is involved in the progression of left ventricular decline, but not one of those in isolation can explain the whole myocardial deterioration. It seems that the remodeling process is the result of the combined effect of the above anatomical and biochemical changes while at the same time left ventricular remodeling in a vicious circle worsens left ventricular function changing the geometry of the left ventricular cavity.

Cardiac remodeling is triggered from significant changes starting at first in the level of cardiac myocytes and later on from the incompetence of the contractile left ventricular cavity apparatus. These mechanical changes consist of the principal events that precipitate cardiac remodeling as many experimental and human studies have demonstrated [50, 51]. In the human cardiac myocyte the changes leading to left ventricular dysfunction, are the decreased α-myosin heavy chain gene expression, the increased β-myosin heavy chain expression, some modifications in cytoskeletal proteins, and the desensitization of β-adrenergic signaling [52-54]. Bringing together, these changes diminish cardiac myocyte contractility and cell shortening, and reduce responsiveness to adrenergic signaling.

The reversibility of the failing cardiac myocyte after β-adrenergic blockade is supported by experimental evidence and studies in human heart failure [55,56]. The functional improvement of the failing cardiac myocyte with β-adrenergic blockade is attributed to the increase in the number of myofilaments inside the diseased cardiac myocytes or to favorable changes in myocardial gene expression involving an increase in α-myosin and a decrease in β-myosin heavy chain mRNA. Also heart failure patients supported with left ventricular assist devices exhibited an improvement of the left ventricular tissue function due to modifications in calcium handling and restoration of the damages in the myocyte cytoskeleton [57, 58]. 

The progressive left ventricular dysfunction and myocardial remodeling is primarily the result of myocardial cell loss with both mechanisms of necrosis and apoptosis. The necrotic cellular death has been connected to an excessive adrenergic stimulation with norepinephrine, angiotensin II and endothelin. In patients with heart failure increased levels of norepinephrine are detected in the blood and myocardial tissue while in experimental heart failure models the myocardial cell death was attributed to increased levels of angiotensin II and endothelin [34, 38]. 

The exact role of apoptosis in the progressive cellular loss in the myocardial tissue is still undefined. It is uncertain whether the contribution of the apoptosis is directed to the whole heart failure process or is only a reality of the advanced stage of heart failure. Experimental and clinical studies are required to determine the exact role of necrosis and apoptosis in patients with mild or moderate heart failure.

The extracellular matrix of the myocardium undergoes some changes such as perivascular fibrosis of the intramyocardial vessels and disproportionate deposition of fibrillar collagen in the place of the lost myocytes [59]. Some experimental studies have shown that the excessive fibrosis is probably related to the inordinate increase of angiotensin II, aldosterone and endothelin [60, 61]. The excessive fibrosis is assumed to guide towards a stiffer myocardium and therefore it is difficult to explain the occurrence of the left ventricular dilation and remodeling during the advance of the heart failure syndrome. It was suggested that matrix metalloproteinases (MMPs) degrade the extracellular collagen contemplating a rearrangement of myocytes in the left ventricular wall followed by a left ventricular cavity dilation and wall thinning [59]. 

The extracellular matrix degradation seems to be a more complex phenomenon that involves cytokines and peptide growth factors that activate the MMPs, and glycoproteins tissue inhibitors of MMPs which also are responsible for matrix regulation by inhibiting the degradation of the collagen cardiac matrix [62]. It appears that many of the myocardial fibrotic changes during the process of left ventricular remodeling and heart failure are irreversible. Bone marrow cells or hematopoietic stem cells failed to differentiate in cardiac myocytes and initiate myocardial regeneration [63], but remains the potentiality of myocardial regeneration through myocardial progenitor cells [64]. 

During the course of heart failure the progressive pathological changes evolved in both myocytes and myocardium are leading towards left ventricular remodeling characterized by left ventricular dilation and sphericity. The remodeled left ventricle demonstrates a spherical geometry with dire mechanical consequences for the failing myocardium. These changes in the geometry and architecture of the remodeled left ventricle aggravate heart failure while the sphericity of the left cavity gives rise to mitral valve papillary muscles dysfunction followed by mitral valve regurgitation [65, 66]. 

The above mechanical changes of left ventricular remodeling are in a position to induce hemodynamic compromise with contractile dysfunction, reduced cardiac output and decreased ejection fraction that lead to heart failure progression. The changed mechanical status of the left ventricle turns the cardiovascular system into a state of decreased sensitivity to adrenergic drive. The overshooting reaction of the compensatory adrenergic mechanisms to the decreased sensitivity also results in a worsening of the clinical situation. 

The mechanical changes of the left ventricular remodeling in some patients are to some degree reversible and possibly preventable. Medical therapy with ACE inhibitors seems to delay the worsening of left ventricular dilation and the increase of left ventricular mass [67]. The use of β-blockers also improves the left ventricular function of some patients with heart failure and left ventricular remodeling [45]. 

The vascular endothelial dysfunction has been a therapeutic target in patients with chronic heart failure based in the interdependence of cardiac and endothelial function [68]. The endothelial dysfunction reduces left ventricular systolic function by increasing systemic vascular resistance while left ventricular mechanical changes can reduce endothelial function by reducing shear stress and vascular nitric oxide bioavailability [68].

Mechanical interventions can reverse or prevent left ventricular remodeling in patients with compromised left ventricular function. Resynchronization therapy improves functional mitral regurgitation [69] and the implantation of a left ventricular assist device reduces left ventricular wall thickness and volume [70]. Some surgical procedures can prevent left ventricular remodeling, such as mitral valve surgery [71], ventricular surgical restoration [72] and left ventricular volume reduction [73].

In this review the biomechanical model is considered as the most probable explanation of the clinical chronic heart failure syndrome, and is viewed as a complex phenomenon with an interaction between left ventricular dysfunction, cardiac cavity remodeling and neurohormonal activation. In the involved mechanical changes participating in the development of heart failure are included the initial (early) mechanical stimulus responsible for cardiac remodeling and neurohormonal activation, and also the progressive mechanical remodeling changes responsible for the persistence and progression of clinical heart failure syndrome [2]. The overexpression of the natriuretic peptide axis and RAAS modules should be considered as an adaptation for survival under the extreme conditions of mechanical deterioration.

In a recent paper, a mathematical model is introduced that predicts the dominant role of remodeling as a compensatory physiological response in chronic heart failure resulting in a normalization of stroke volume [74]. In the same paper is proposed the following hypothesis: 1) The heart failure syndrome in the pre-compensation (un-remodeled) stage is a consequence of an initial decline of the stroke volume due to reduced long-axis shortening, while the compensatory remodeling ventricular dilation normalizes stroke volume and 2) the presence of concentric left ventricular hypertrophy is the main factor that determines whether HFrEF (heart failure with a reduced ejection factor and absence of hypertrophy) or HFpEF (heart failure with a preserved ejection factor and presence of concentric hypertrophy) would be developed [74]. In patients with HFpEF there is systolic left ventricular impairment as measured by the reduction of the longitudinal axis in both diastole and systole, and calculated by the mitral annular excursion in systole (S_LAX_) and early diastole (E_LAX_) which were lower in the patients with diastolic heart failure [75]. Left ventricular mitral annulus velocities measured by tissue Doppler imaging in early diastole is a predictor for cardiac mortality compared with clinical data and other standard echocardiographic measurements in patients with a variety of cardiac diseases and ventricular function [76]. Systolic abnormalities were identified in patients with HFpEF with the use of tissue Doppler imaging and the detection of significantly lower values of peak regional myocardial sustained systolic(S_M_) and early diastolic (E_M_) velocities in patients with HFrEF, HFpEF and isolated diastolic dysfunction [77]. The Doppler derived ratio of early transmitral flow velocity to early diastolic mitral annular velocity (E/E^’^) is useful to assess left ventricular filling pressure while a persistent elevation of this ratio may be a prognostic factor of diastolic heart failure in patients with HFpEF independent of left ventricular hypertrophy [78]. In patients with HFpEF the elevated E/E^’^ ratio after optimized medical therapy is predictive of cardiac events [79]. In patients with ischemic heart disease a ratio E/E^’^ greater than 15, ejection fraction less than 50%, and severe functional mitral regurgitation were independent echocardiographic predictors of cardiac events [80]. In patients with impaired left ventricular systolic function an E^’^<3 cm/s was associated with a significantly excess mortality, and this measurement added incremental prognostic value to E/E^’^ >15 [81].

In the end-stage of heart failure the persistent mechanical remodeling changes are self-determined probably without neurohormonal dependence operating until the end of the disease. In this stage it is improbable for the neurohormonal activation to have a sustained and effective inotropic performance and therefore the only compensatory mechanism that remains is the left ventricular remodeling. The compensatory mechanisms of left ventricular remodeling and neurohormonal activation are not sufficient to counterbalance effectively the initial mechanical left ventricular changes while the progressive mechanical remodeling changes that occur during the end-stage of the left ventricular dysfunction are deleterious and unopposed by any other compensatory mechanisms. In a sense significant left ventricular dysfunction begets progressive ventricular remodeling, that in turn begets progressive left ventricular dysfunction. The end-staged left ventricle represents a terminally remodeled and noncompliant cavity with low contractility and function.

## EARLY ENDOVASCULAR AND INTRACARDIAC MECHANICAL CHANGES AND HEART FAILURE 

The mechanosensation is an old biological process to be found in all forms of life. In mechanotransduction the mechanical effect is closely related and affects the transduction systems of vasopeptides, hormones, sensing receptors, ionic fluxes and other autocrine/paracrine mechanisms.

The endothelium of arteries and veins is constantly exposed to hemodynamic forces due to the pulsatile nature of blood pressure and flow. Therefore the endothelium is permanently detecting various biomechanical forces (i.e., cyclic stretch and shear stress), which are translated into intra- and extracellular signals [82]. The biomechanical forces applied to endothelial cells are responsible for the production of cytokines, enzymes and growth hormones, and are mediating inflammatory responses, hemostasis and vascular smooth muscle cell contraction [83]. An equilibrium exists between shear stress and endothelial function. A reduced shear stress or low wall shear stress gradient are considered proatherogenic and prothrombotic increasing the possibility for atherosclerosis, while elevation of shear stress induces arteriogenesis [84, 85]. The protective effect of shear stress is based to the local release of nitric oxide that reduces leucocyte adhesion, vascular smooth muscle cell proliferation and endothelial permeability [86].

The Kruppel-like factor (KLF2) is a transcriptional inhibitor of endothelial mediated inflammation [87]. The KLF2 is upregulated by shear stress and its role is significant in hemodynamic regulation in response to arterial shear stress. Loss of KLF2 results in heart failure of high cardiac output state in both zebrafish and mice, indicating loss of peripheral resistance [88]. In experiment models of murine cardiac hypertrophy and in human heart failure increased expression of early genes and embryonic markers was found [89]. 

The biomechanical stress is speculated as an early and most significant stimulus for cardiac hypertrophy and heart failure. The gp130 cytokine receptor plays a significant role in myocyte survival pathway in the transition to heart failure. During aortic pressure overload, in mice with ventricular restricted knockout of the gp139 cytokine receptor, was described a rapid onset of dilated cardiomyopathy and massive myocyte apoptosis in contrast to control mice that showed compensatory hypertrophy [90]. The activation of tyrosine phosphorylation of STAT3, a molecule in the gp130 pathway, appears early after the biomechanical stimulus of pressure overload and this activation was not present in the gp130 knockout mice. 

It is hypothesized that in the adult the early hemodynamic cavity stresses of left ventricular dysfunction induce ventricular endothelial dysfunction and remodeling. Probably that follows the embryonic model of flow-structure interactions that influence the process of cardiac development [91]. The intracardiac fluid forces play a significant role in cardiac morphogenesis and in human heart failure and disease as it is described in quantitative *in vivo* analyses of intracardiac flow forces in zebrafish embryos [92]. The recognition of the precise signaling pathways that mediate the stress activated responses consists of one of the most important questions in cardiovascular biology [93]. The analysis of potential regulatory pathways that change the growth of mature myocardial cells in response to stress, is advanced through culture of adult cardiac myocytes. The study of the subcellular mechanism of the signal pathways is restrained when whole animals are employed due to the multicellular consistency and the neurohormonal interactions of the whole myocardium. The mechanical load through stimulation of α and β adrenergic receptors, is the predominant stimulus to growth in the adult myocardial cell hypertrophy with two processes, protein synthetic capacity and myofibrillar organization [94]. The β adrenergic activation increases cellular hypertrophy through its inotropic properties, in contrast to α adrenergic agonists that increase the synthesis of contractile proteins but they are deprived of the property of myofibril organization.

Myocardial biomarkers like natriuretic peptides are considered useful biochemical substances in the diagnosis of patients with heart failure. The natriuretic peptides ANP and BNP are secreted in response to increasing cardiac wall tension and/or circulating neurohormones. The blood levels of ANP and BNP are increased in patients with left ventricular dysfunction as well in patients with preserved ejection fraction. In patients with systolic heart failure the BNP blood levels are directly related to wall stress, ejection fraction and functional failure classification [95]. In women the natriuretic peptide levels are more elevated probably caused by the higher wall stress in the smaller female cardiac cavity [96]. Detailed early mechanical data regarding brain natriuretic peptide activation in the context of cavity or vascular dysfunction, are limited in cultured cardiac myocytes through signal regulated kinase pathways. One of the earliest and most reliable marker of ventricular cardiac myocyte hypertrophy is the activation of BNP gene promoter activity. It is demonstrated that signaling mechanisms are underlying the strain dependent BNP secretion and BNP gene transcriptional activity in neonatal rat myocyte cultures [97]. Also it was demonstrated that the application of mechanical strain in cultured cardiac myocytes activated the BNP gene promoter through the p38 mitogen-activated protein kinase (MAPK) and the extracellular signal regulated kinase (ERK) pathways. In addition the endothelin-dependent and endothelin-independent components of the transcriptional response to mechanical strain use similar signal transduction pathways [98, 99]. In another study was tested the hypothesis that cyclic mechanical stretch specifically increases engineered early embryonic cardiac tissue (EEECT) proliferation mediated by p38MAPK activity. The findings imply that the embryonic cardiac cells proliferation is positively regulated by mechanical stretch and negatively regulated by p38MAPK inhibition [100]. The mixed-lineage kinase (MLK1-3) signaling pathway regulates stress response in cardiac myocytes via nuclear effectors. In an experimental study it was found that inhibition of the MLK1-3 signalling pathway suppresses the activity of key nuclear factors in cardiac hypertrophy, and reduces the atrial natriuretic peptide secretion and the activation of BNP gene transcription [101]. 

Shear stress demonstrates an atheroprotective role through downregulation of angiotensin type 1 receptors (AT_1_R), as it is well known that angiotensin II is proinflammatory and proatherosclerotic. This was demonstrated in an immunohistochemical analysis in the aortic arch of transgenic mice where a pronounced expression of AT_1_R was found in the inner atheroprone regions of the aortic arch, characterized by disturbed or oscillatory shear stress, but not in the outer aortic arch exposed to high shear stress [102]. In the same paper it was also demonstrated that in cultured human umbilical vein endothelial cells, laminar shear stress induced a decrease in AT_1_R protein expression.

**Fig. (2):**
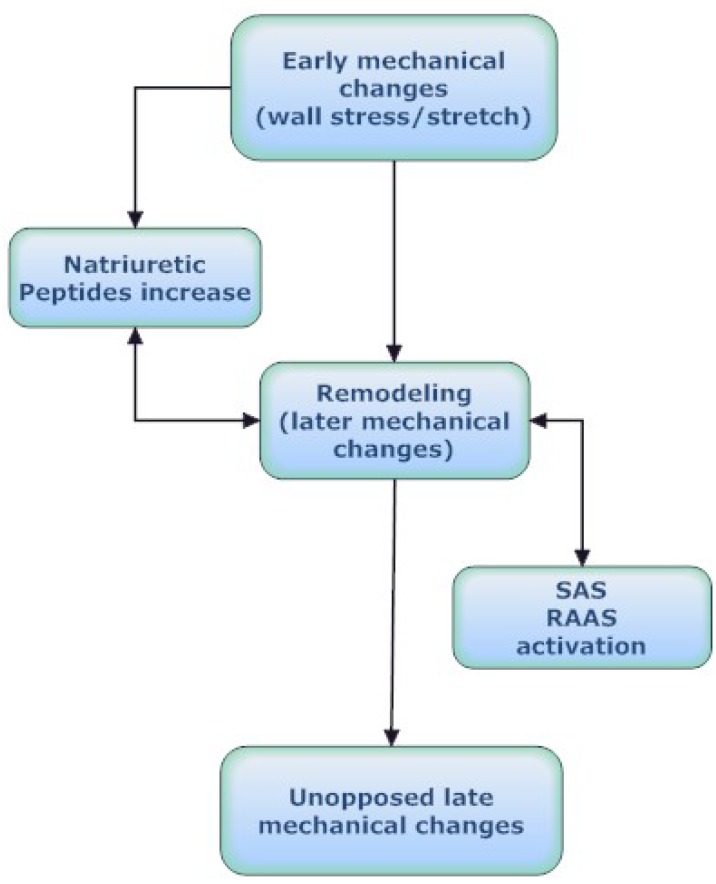
Interdependence between myocardial dysfunction, cardiac remodeling and neurohormonal activation.

How can the early mechanical effects be translated into clinical phenotypes? The impact of the early mechanical changes on clinical outcome in heart failure patients remains to be established in a future study comparing initial mechanical changes with the earliest neurohormonal activation. It is significant from a clinical perspective the design of a trial with sufficient power to discern meaningful interactions between early mechanical effects and neurohormonal activation in order to improve our understanding of the later clinical outcome (Fig. **2**).

The transgenic technology queries the validity of the wall-stress hypothesis that both cardiac hypertrophy and fetal reprogramming were a necessary adaptive response to mechanical load. It is suggested that the progression of heart failure depends on different mechanisms triggered by new molecular rearrangements. The above position is not absolutely valid and it is not necessary to reconsider the wall-stress hypothesis [103]. Swynghedauw *et al* (103) give several reasons supporting the wall-stress hypothesis: 1) Pure mechanical overload that is not accompanied by a neurohormonal reaction is a rather rare phenomenon and therefore the coexistence of the two processes obscures the significance of the mechanical initial factor. 2) The transgenic hypothesis is impossible to reproduce the entire cascade that follows the mechanical stress. 3) During cardiogenesis the different transcription factors operate synergistically in a combinatorial way that is not documented in cardiac hypertrophy. They support the hypothesis that cardiac hypertrophy results from an initial mechanical effect and a re-expression of the fetal program, and during clinical conditions in humans the effects of mechanics are modified by senescence, obesity, diabetes and ischemia [103]. 

## CLINICAL PHENOTYPES IN HEART FAILURE

Phenotype in biology is considered any observable characteristic of an organism that includes morphology, biochemical properties, physiological functions, and behaviors. As phenotypic data from systems biology perspective are examined network functional states such as fluxomic data that give information about the actual flux distributions in a network [104]. The complex process of network functional states, that leads from genes to proteins, and from modular functional units to clinical models (clinical phenotypes) provides a comprehensive picture of heart failure progression. The mechanistic model of heart failure predicts better than the other models the deterioration of heart failure which after a point progresses independently of the neurohormonal status [2] (Fig. **2**). 

Human heart failure is a syndrome exhibiting multiple clinical phenotypes implicating a number of common pathophysiological, biochemical and molecular mechanisms. The clinical phenotypes are marked by multiorgan dysfunction due to the upregulation or downregulation of the above mechanisms. It is important from experimental and clinical perspective to define these phenotypes and to schedule a therapeutic strategy of multifaceted heart failure targeting to a more personalized therapy. 

The described heart failure models represent clinical phenotypes that can be studied using the current available data from the advanced fields of genomics, proteomics and experimental model systems [74]. Recently have been clarified the mechanical disadvantages produced by left ventricular remodeling and the significance of the regulatory systems of natriuretic peptide axis and RAAS. These regulatory systems are considered clinical functional modules that act both as compensatory mechanisms modulating left ventricular function and as destructive agents of heart failure progression promoting or inhibiting the deleterious effects of cardiac remodeling [9].

Therefore the neurohormonal dysfunction initially compensates cardiac hemodynamic changes and in a later stage becomes toxic to the myocardium reducing myocardial contractility and increasing the deleterious processes of cardiac remodeling.

In conclusion the systems biology analysis of heart failure will increase our understanding of the biological and mechanical basis of the disorder. The progressive nature of the mechanical model of the heart failure from early mechanical changes to left ventricular remodeling and the modular analysis of the neurohormonal homeostatic regulation will produce a comprehensive analysis of the disease process. The elucidation of the complex heart failure disease will also provide the material for further development of new therapeutic strategies.
